# Flux Synthesis of A‐site Disordered Perovskite La_0.5_M_0.5_TiO_3_ (M═Li, Na, K) Nanorods Tailored for Solid Composite Electrolytes

**DOI:** 10.1002/advs.202408805

**Published:** 2024-11-25

**Authors:** Tao Wang, Jiyoung Ock, X. Chelsea Chen, Fan Wang, Meijia Li, Matthew S. Chambers, Gabriel M. Veith, Lauren B. Shepard, Susan B. Sinnott, Albina Borisevich, Miaofang Chi, Amit Bhattacharya, Raphaële J. Clément, Alexei P Sokolov, Sheng Dai

**Affiliations:** ^1^ Chemical Sciences Division Oak Ridge National Laboratory Oak Ridge TN 37831 USA; ^2^ Department of Chemistry Institute for Advanced Materials and Manufacturing University of Tennessee Knoxville TN 37996 USA; ^3^ Department of Materials Science and Engineering The Pennsylvania State University University Park PA 16802 USA; ^4^ Department of Chemistry The Pennsylvania State University University Park PA 16802 USA; ^5^ Institute for Computational and Data Sciences The Pennsylvania State University University Park PA 16802 USA; ^6^ Materials Research Institute The Pennsylvania State University University Park PA 16802 USA; ^7^ Center for Nanophase Materials Sciences Oak Ridge National Laboratory Oak Ridge Tennessee 37831 USA; ^8^ Materials Department and Materials Research Laboratory University of California Santa Barbara CA 93106 USA

**Keywords:** disordered structure, flux synthesis, molten salts, nanomaterials, solid composite electrolyte

## Abstract

Inorganic fillers play an important role in improving the ionic conductivity of solid composite electrolytes (SCEs) for Li‐ion batteries. Among inorganic fillers, perovskite‐type lithium lanthanum titanate (LLTO) stands out for its high bulk Li^+^ conductivity on the order of 10^−3^ S cm^−1^ at room temperature. According to a literature survey, the optimal LLTO filler should possess the following characteristics: i) a single‐crystal structure to minimize grain boundaries; ii) a small particle size to increase the filler/polymer interface area; iii) a 1D morphology for efficient interface channels; and iv) cubic symmetry to facilitate rapid bulk Li^+^ diffusion within the filler. However, the synthesis of single crystal, 1D LLTO nanomaterials with cubic symmetry is challenging. Herein, a flux strategy is developed to synthesize La_0.5_M_0.5_TiO_3_ (LMTO, M═Li, Na, and K) single‐crystal nanorods with an A‐site‐disordered, cubic perovskite phase. The flux media promotes the oriented growth of nanorods, prevents nanorods from sintering, and provides multiple alkali metal ion doping at M sites to stabilize the cubic phase. SCEs compositing the Li^+^‐conducting LMTO nanorods as fillers and poly[vinylene carbonate‐*co*‐lithium sulfonyl(trifluoromethane sulfonyl)imide methacrylate] matrix exhibit more than twice the conductivity of the neat polymer electrolyte (30.6 vs 14.0 µS cm^−1^ at 303 K).

## Graphical abstract

Single crystal La_0.5_M_0.5_TiO_3_ (LMTO, M═Li, Na, and K) nanorods with an A‐site‐disordered, cubic perovskite phase are synthesized by a flux strategy. The flux media promotes the oriented growth of nanorods, prevents nanorods from sintering, and provides multiple alkali metal ion doping at M sites to stabilize the cubic phase.

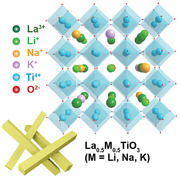

## 1 Introduction

There has been considerable effort invested into researching solid‐state electrolytes because of their wide electrochemical stability windows, high‐temperature resistance, and good mechanical properties compared to conventional liquid electrolytes.^[^ ^1^, ^2^, ^3^ ^]^ Among the well‐known inorganic solid electrolytes, the perovskite (ABO_3_)–type lithium lanthanum titanate (LLTO) has a very high bulk lithium (Li) ionic conductivity on the order of 10^−3^ S cm^−1^ at room temperature.^[^ ^4^ ^]^ However, polycrystalline LLTO pellets suffer from low macroscopic ionic conductivity on the order of 10^−5^ S cm^−1^ due to the high grain boundary impedance.^[^ ^5^, ^6^ ^]^ Single‐crystal (SC) LLTO could solve the grain boundary issues and exhibit high conductivities of 4.0 × 10^−3^ S cm^−1^ at 300 K,^[^ ^7^ ^]^ but the scaled production of SC pellets is still limited due to the complexity of the operation.^[^ ^8^ ^]^ Compared to inorganic solid electrolytes, solid polymer electrolytes have advantages including large‐scale processability, high tolerance to mechanical deformation, and good adhesion to electrodes.^[^ ^2^, ^9^, ^10^, ^11^, ^12^, ^13^ ^]^ The drawbacks of polymer electrolytes are their low ionic conductivities below their glass transition temperatures and poor electrochemical stability.^[^ ^2^, ^14^ ^]^ To mitigate the drawbacks of individual LLTO and polymer electrolytes, solid composite electrolytes (SCE) stand out by incorporating LLTO fillers in polymer electrolytes to balance ionic conductivity, mechanical strength, and electrochemical stability.^[^ ^15^, ^16^, ^17^, ^18^ ^]^

**Figure 1:**
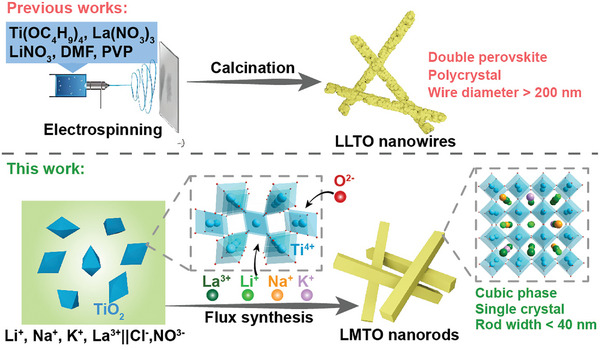
Comparison of a typical electrospinning process for LLTO nanowire synthesis and the flux synthesis approaches developed in this work for LMTO nanorods.

Since the superior performance of LLTO/polymer SCE is mainly from additional filler/polymer interfaces and the high bulk Li^+^ conductivity of fillers, the morphology (size and shape) and crystalline phases of LLTO fillers are the determining factors underpinning conductivity enhancements in SCE.^[^ ^16^, ^19^, ^20^ ^]^ LLTO nanofillers could provide a large specific surface area to maximize the LLTO/polymer interfacial volume.^[^ ^17^, ^18^, ^21^ ^]^ Regarding the shape of the LLTO fillers, Cui et al. reported that nanowire fillers with a high aspect ratio provided continuous ionic transport pathways over much longer distances as compared to nanoparticle fillers.^[^ ^22^, ^23^ ^]^ 1D nanowires synthesized by electrospinning, on the other hand, were polycrystalline, containing numerous grain boundaries, where the huge grain boundary resistance limited the ionic conductivity of ceramic‐polymer composite electrolytes (**Figure** **1**).^[^ ^24^, ^25^, ^26^ ^]^ According to the literature, the huge grain boundary resistance of LLTO nanofibers synthesized through electrospinning limited the ionic conductivity of ceramic‐polymer composite electrolytes. In their work, hydrogen treatment was used to create oxygen vacancies in the LLTO nanofibers, which reduced the activation energy of Li‐ion transport along intra‐grains and inter‐grains, leading to an improvement in the ion conductivity of the composite electrolyte.^[^ ^26^ ^]^ A better choice is SCs, which could eliminate grain boundaries and facilitate Li diffusion within the fillers.^[^ ^27^ ^]^ Regarding the preferred crystalline phases, the A‐site disordered cubic (space group: *Pm‐*3*m* ) or the tetragonally‐distorted pseudocubic (space group: *P*4*/mmm*) structures of LLTO have higher ionic conductivities than the tetragonal double perovskite (space group: *P*4*/mmm*), cubic, hexagonal^[^ ^28^ ^]^ and orthorhombic phases.^[^ ^29^, ^30^ ^]^ The main reason for the low conductivity of the other phases is the uneven distribution of Li, La, and vacancies along the C‐axis direction.^[^ ^31^ ^]^ Overall, SC LLTO nanorods (NRs) with a cubic structure should be the best LLTO filler for SCE. However, the synthesis of SC LLTO nanorods with a cubic phase is still challenging due to the tradeoff between the need for low‐temperature synthesis to prevent nanoparticles from sintering and the high tetragonal–cubic phase transition temperature (> 1423 K) for LLTO.^[^ ^32^, ^33^, ^34^ ^]^

Targeting mild experimental conditions for the synthesis of high‐temperature phases, the ionothermal synthesis strategy stands out, using molten salts as both solvent and structure‐directing agent during crystallite growth.^[^ ^35^, ^36^ ^]^ The molten salt synthesis method can facilitate crystal growth with improved phase purity and particle homogeneity for a controllable morphology synthesis.^[^ ^37^ ^]^ Perovskite BaTiO_3_ nanorods were synthesized by Huang et al., using molten potassium chloride (KCl) and sodium chloride (NaCl) as flux media at 973 K.^[^ ^38^ ^]^ The insoluble titanium oxide particles in molten NaCl‐KCl reunited into rod‐like particles, acting as templates for the further growth of nanorods.^[^ ^39^, ^40^ ^]^ The ionicity of molten salts supplies strong Coulombic interactions among the constituent ions and the inherent nonvolatility, which allows for efficient mass transport in a so‐called flux in the production of high‐temperature phase products under ambient pressure.^[^ ^41^ ^]^ Herein, we use the flux synthesis strategy to prevent sintering while also facilitating the stabilization of the high‐temperature cubic phase and report a flux conversion of commercial P25 type titanium dioxide (P25‐TiO_2_) nanoparticles to SC La_0.5_M_0.5_TiO_3_ (named as LMTO‐x, M═Li, Na, and K, x is the flux temperature in Kelvin) NRs in molten salts (Figure 1). In the flux synthesis of SC LMTO NRs, KCl and NaCl were used as the high‐temperature flux medium. La(NO_3_)_3_ and LiNO_3_ were used to lower the melting point of the flux medium and to supply La and Li sources of LMTO, respectively. P25‐TiO_2_ nanoparticles with mixed rutile and anatase phases were used as the Ti source. SC LMTO NRs have an A‐site disordered, cubic, perovskite phase, SC structure, and D50 rod with (the average rod width) below 40 nm. The SCE comprising LMTO‐1073 NRs as a filler exhibited more than twice the conductivity of the pure polymer electrolyte and of the SCE using commercial LLTO powder as a filler.

## 2 Results and Discussion

### 2.1 Morphology and Structure of LMTO Nanorods

**Figure 2:**
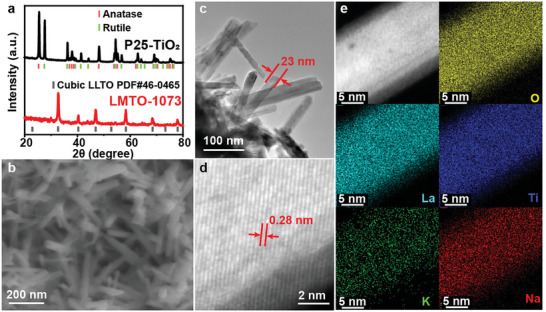
a) XRD patterns of P25‐TiO_2_ and LMTO‐1073; b) SEM image of LMTO‐1073; c) TEM image of LMTO‐1073; d) HAADF‐STEM image of LMTO‐1073; e) STEM images and EDS element mapping of LMTO‐1073.

As shown in Figure 1, the molten salts containing four cations (Li^+^, Na^+^, K^+^, and La^3+^) and two anions (Cl^−^, and NO_3_^−^) were used as the flux medium for the conversion of TiO_2_ into LMTO nanorods. The flux medium comprises a binary KCl‐NaCl salt as the main body. LiNO_3_ and La(NO_3_)_3_ were introduced to KCl‐NaCl to serve as Li and La sources and lower the melting point of the flux medium (Figure S1, Supporting Information). The crystalline phases of P25‐TiO_2_ and LMTO were identified by powder X‐ray diffraction (XRD). As shown in **Figure** **2**a, P25‐TiO_2_ exhibited a mixture of anatase and rutile phases. After flux synthesis at 1073 K, the XRD pattern of LMTO‐1073 matches well with the cubic LLTO phase (PDF#: 46–0465). The morphology of LMTO‐1073 was characterized by a scanning electron microscope (SEM) and transmission electron microscopy (TEM). The SEM image of LMTO‐1073 reveals nanorods with a D50 rod width of 33 nm (Figure 2b). The TEM image of LMTO‐1073 reveals SC rods (Figure 2c) and the high‐angle annular dark‐field scanning transmission electron microscopy (HAADF‐STEM) image (Figure 2d) reveals a clearly continuous lattice fringe corresponding to the (110) planes. Inductively coupled plasma optical emission spectroscopy (ICP‐OES) results show that the atomic ratio of Li:Na:K:La:Ti is ≈0.11(1):0.243(5):0.02(1):0.431(7):1 in LMTO‐1073 (Table S1, Supporting Information), indicating Na and K doping on the A sites of the LMTO structure. The very low amount of K incorporated into the products in comparison to the amount of Na is due to the higher ionic radius of K^+^ with respect to La^3+^.^[^ ^42^ ^]^ The uniform distribution of Na, K, La, Ti, and O elements was validated by the energy dispersive X‐ray spectroscopy (EDS) element mapping (Figure 2e). It has been shown that increasing the entropy of a system by increasing the number of elements on the same site can stabilize high‐symmetry phases,^[^ ^43^, ^44^ ^]^ such as with disordered rock salts^[^ ^45^ ^]^ and with other perovskites.^[^ ^46^ ^]^ Rietveld refinement of the powder XRD pattern was performed to determine the effect of Na and K doping on the polymorphic form of LMTO and the results are discussed in the supporting information. Since TiO_2_ and La_2_Ti_2_O_7_ impurities exist in LMTO, the ratio of La^3+^ within the LMTO‐1073 sample should be different from the ratio obtained through ICP. A final refinement was performed with the cubic *Pm‐*3*m* polymorph with Li, Na, K, and La at A‐sites, with the occupancies of Li, Na, K, Ti, and O fixed to those determined through ICP analysis (Li_0.11(1)_Na_0.243(5)_K_0.02(1)_La_0.431(7)_TiO_2.82(2)_) while the occupancy of La was allowed to freely refine. The A‐site cations shared an isotropic atomic displacement parameter (ADP), while separate ADPs were used for Ti and O, resulting in a La occupancy of 0.491(13). Since La^3+^ has a much larger X‐ray form factor factor than the other elements in LMTO, this is only an approximate value for the cation distribution. Additionally, the size broadening of the LMTO crystallites was modeled using a Voigt function,^[^ ^47^ ^]^ resulting in a fit of R_wp_ = 4.841%, χ^2^ = 1.473, and an integral breadth‐based volume‐weighted column height L_vol_ of 39(5) nm. It should be noted that refinement using the pseudocubic *P*4/*mmm* phase provides equally good fits (R_wp_ = 4.880%, χ^2^ = 1.484) and *R*‐3*c* (R_wp_ = 5.025%, χ^2^ = 1.527), as the broad peaks in the powder pattern make it difficult to resolve which polymorph is present. The reason for selecting the cubic polymorph is discussed in more detail in the Supporting Information. The Rietveld plots using the cubic polymorph are shown in Figure S2a (Supporting Information). Observations of the nanowires using diffractogram analysis of Annular Bright Field (ABF) images in Scanning Transmission Electron Microscope (STEM) did not reveal any additional reflections indicative of orthorhombic or tetragonal distortions of the wires (Figure S3a,b, Supporting Information). Precise tilting of the samples is hindered by beam sensitivity of the Li‐and vacancy‐filled sublattice and overlaps due to sample embedding, however, multiple areas can be assigned *Pm‐3* *m* orientation that closely matches angles and values of observed spacings. Selected area electron diffraction (SAED) in TEM mode (Figure S3c, Supporting Information) similarly did not reveal any reflections inconsistent with the *Pm‐3* *m* lattice. We note that there are 8.1(6)% TiO_2_ and 9.0(5)% La_2_Ti_2_O_7_ impurity phases present in the sample. To decrease the La_2_Ti_2_O_7_ impurity in the LMTO product, the flux temperature was increased from 1073 to 1173 K to obtain LMTO‐1173. In this case, Rietveld refinements indicated successful removal of the La_2_Ti_2_O_7_ impurity, resulting in a composition of 90.4(4)% of LMTO and 9.6 (4)% of TiO_2_. ICP analysis indicated that the atomic ratio of Li:Na:K:La:Ti is ≈0.01:0.27:0.01:0.38:1 in LMTO‐1173. The very low amount of Li in LMTO‐1173 is due to the Li loss during the high‐temperature synthesis.^[^ ^48^ ^]^ Based on the results above, SC LMTO NRs (LMTO‐1073 and LMTO‐1173) with A‐site disordered cubic perovskite phase were successfully synthesized using the flux synthesis strategy.

### 2.2 Growth Mechanism of LMTO Nanorods in Molten Salts

**Figure 3:**
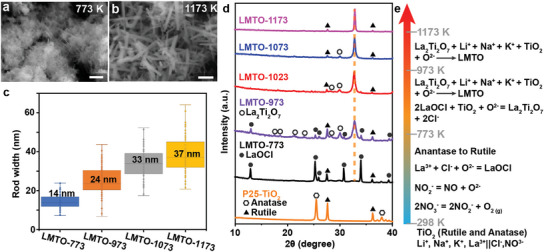
a,b) SEM images of LMTO‐773 (a) an LMTO‐1173 (b); c) Rod width distributions of LMTO‐x; the solid boxes are interquartile ranges and the D50 rod width is marked on each solid box. The rod width distribution of LMTO‐773 was obtained based on the TEM image in Figure S5 (Supporting Information). d) XRD patterns of P25‐TiO_2_ and LMTO‐x. Scale bars in a and b are 200 nm. The orange dash line in d indicates the mean diffraction peak from the cubic LMTO phase. e) Illustration of main reactions in different temperature ranges.

To shed light on the growth mechanism of LMTO, thermogravimetric analysis (TGA) and differential thermal analysis (DTA) were performed on the reaction system to analyze the decomposition and phase transition process. As shown in Figure S1 (Supporting Information), the TGA curve of the reaction mixture has two significant weight loss stages with onset temperatures equal to 640 and 939 K, corresponding to the decomposition of nitrates and chlorides (e.g., LaOCl), respectively. A couple of endothermic peaks appear below 473 K in the DTA curve, corresponding to the evaporation of adsorbed water and water of crystallization. Before the decomposition of the nitrates, the endothermic peaks at 579 K, where no weight loss is observed in the TGA curve, presumably arise from the melt of salts. The low melting point of the molten salts enables an effective flux process when heating the reaction mixture above the melting point. Though nitrates decompose at ≈640 K, the residual chlorides NaCl/KCl can still work as a flux media up to 1073 K.^[^ ^49^ ^]^ In addition, variable temperature syntheses were conducted over a wide temperature range from 773 to 1173 K. As shown in **Figures** **3**a and S5 (Supporting Information), nanorods with a D50 rod width of 14 nm are formed on TiO_2_ nanoparticles in LMTO‐773. The XRD pattern of LMTO‐773 reveals strong peaks from LaOCl and rutile (Figure 3d), indicating the decomposition of La(NO_3_)_3_ and the conversion of anatase to rutile during heating. The broad hump at 32.7° corresponds to nanosized LMTO. Increasing the flux temperature from 773 to 973 K results in a much sharper diffraction peak at 32.7° and additional peaks from La_2_TiO_7_. Those peaks from the LaOCl phase disappeared upon increasing the flux temperature to 1023 K. The peak intensity ratio of La_2_TiO_7_ at 29.9° to LMTO at 32.7° decreased from 0.32 to 0.07 upon increasing the flux temperature from 973 to 1073 K. Meanwhile, the D50 rod width increased from 14 to 33 nm (Figure 3c). Peaks from La_2_TiO_7_ disappeared in the XRD pattern of LMTO‐1173 (Figure 3d). Based on the temperature‐dependent synthesis results and the TGA/DTA data, the proposed reaction sequence is depicted in Figure 3e. The salts melt at 579 K and then the reaction begins with the decomposition of NaNO_3_ to nitric oxide, oxygen gas, and Na_2_O at 640 K, and the formation of LaOCl below 773 K. Then, LaOCl decomposes above 939 K and reacts with TiO_2_ to generate La_2_Ti_2_O_7_ until complete decomposition of LaOCl at 1023 K. Meanwhile, La_2_Ti_2_O_7_ incorporates with alkali metal cations from the molten salts to generate LMTO. As the temperature increases to 1173 K, La_2_Ti_2_O_7_ is fully converted to LMTO.

**Figure 4:**
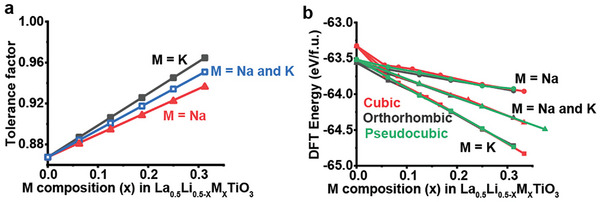
a) Tolerance factor values calculated for different M compositions in LMTO. Note that a tolerance factor of 0.9 or greater is generally accepted as the cubic perovskite phase range. b) Energy per formula unit at 0 K for cubic (red), orthorhombic (black), and pseudocubic (green) LMTO phases as it varies with M═Na (circles), M═K (squares), and M═Na and K at equal compositions (triangles).

Density Functional Theory (DFT) calculations were used to show that doping with K^+^ and Na^+^, which are larger than Li^+^, on the A‐site can help stabilize the cubic LMTO phase. Average Ti─O─Ti bond angles with varying Na and/or K doping were obtained from DFT geometry relaxations of the orthorhombic (space group: *Pnma*) La_0.5_Li_0.5‐x_M_x_TiO_3_ structure. As the dopant content increases, the average Ti─O─Ti bond angle increases, indicating that doping with K and Na can favor a higher symmetry structure with less TiO_6_ rotation (Figure S7, Supporting Information). Tolerance factors were calculated for various Na and/or K doping compositions in LMTO. For a given choice of ionic radii, cubic perovskite coordination environments were considered.^[^ ^50^ ^]^ For a cubic perovskite phase to be favored, a tolerance factor in the range of 0.9–1.0 is needed. As seen in **Figure** **4**a, Li_0.5_La_0.5_TiO_3_, without any Na and/or K doping, has a tolerance factor outside the cubic perovskite range. As the Na and/or K dopant content increases in LMTO, the tolerance factor increases to eventually reach a value above 0.9, indicating that doping with the larger Na and K cations can stabilize a cubic LMTO phase. Figure 4b shows the relative DFT energy per formula unit of the cubic, orthorhombic, and pseudocubic LMTO phases as they relate to Na and/or K dopant concentrations. In the absence of Na or K doping, the cubic phase is higher in energy than the lower symmetry orthorhombic and pseudocubic phases. As the concentration of Na and K dopants increases, the energy gap between the cubic LMTO phase and the orthorhombic and pseudocubic phases decreases significantly. However, it should be noted that polymorph selectivity in LLTO is complicated and cannot always be explained by thermodynamics alone.^[^ ^33^ ^]^ Nevertheless, the DFT calculations do not disagree with the experimental results. This shows that doping with Na and/or K on the A‐sites in the LMTO perovskite structure can stabilize a higher symmetry cubic phase. This is consistent with the fact that the Li^+^, Na^+^, K^+^, La^3+^||Cl^−^, NO_3_^−^flux medium is critical for the synthesis of A‐site disordered SC LMTO NRs, ensuring the even reaction of reactants to minimize side reactions, promoting the crystallization of LMTO, and controlling the directional growth of the nanorods.

### 2.3 Li‐ion Dynamic in LMTO Nanorods and SCEs

**Figure 5:**
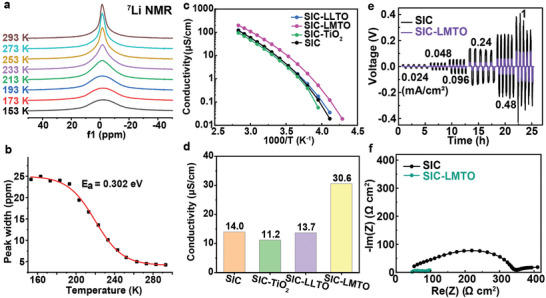
a) Temperature‐variable static ^7^Li NMR spectra for LMTO‐1073; b) Full‐width at half maximum of the ^7^Li spectral line shape versus temperature from the results of temperature‐variable static ^7^Li NMR tests; c) Temperature‐variable conductivities of SIC‐LLTO, SIC‐LMTO, SIC‐TiO_2_, and SIC; d) Conductivities of SIC‐LLTO, SIC‐LMTO, SIC‐TiO_2_, and SIC at 303 K; e) Li symmetrical cell cycling at different current densities with SIC and SIC‐LMTO membranes; f) Impedance spectra of the membranes before cycling.

The ion transport properties of LMTO were investigated in its as‐prepared state by solid‐state NMR, which allows for bulk ion dynamics to be probed without being impacted by external factors such as the quality of the solid‐state electrolyte pellet. Static NMR spectra of the quadrupolar ^7^Li nucleus (spin quantum number I = 3/2) were collected at a Larmor frequency of ω_0_/2π = 155.38 MHz. The NMR spectral line shape is highly dependent on the local and long‐range Li‐ion diffusion processes, and its evolution with temperature can provide information concerning the rate of Li‐ion hopping. In general, the shaper ^7^Li signals indicate higher Li^+^ diffusion dynamics. As shown in **Figure** **5**a, one symmetric peak is observed in the ^7^Li NMR spectrum collected on LMTO‐1073 at 293 K. ^7^Li NMR spectra were collected at various temperatures to determine the activation energy for Li^+^ diffusion. As shown in Figure 5b, the NMR linewidth as a function of temperature for LMTO‐1073 displays a typical motional narrowing curve, with broadened spectra at low temperatures due to residual quadrupolar interactions and ^7^Li‐^7^Li spin dipolar interactions. The observed narrowing of the spectral line shape with increasing temperature is due to the motional averaging of these anisotropic interactions. The activation energy barrier for Li diffusion can be estimated using the empirical equation from Waugh and Fedin: Ea = 1.617 × 10^−3^ × T_c_/K, with T_c_ denoting the onset temperature of motional narrowing.^[^ ^51^ ^]^The onset temperature of line narrowing for LMTO is ≈187 K, corresponding to a low activation energy barrier of 0.302 eV, confirming the fast Li^+^ dynamic properties of bulk LMTO.

LMTO‐1073 nanorods were dispersed in the monomers vinyl ethylene carbonate (VEC) and lithium sulfonyl(trifluoromethane sulfonyl)imide methacrylate (LiMTFSI) of a single‐ion‐conducting (SIC) polymer, to enable the in situ polymerization in presence of the LMTO‐1073 nanorods to form SIC‐LMTO composite electrolyte. The SIC polymer rather than a dual‐ion‐conducting polymer host was selected as the polymer matrix because SIC systems minimize space charge layers at the polymer‐ceramic interface (since both the polymer and ceramics are single‐ion or near single‐ion conductors).^[^ ^52^ ^] 6^Li NMR spectra obtained at room temperature (303 K), 18.8 T, and at 30 kHz magic angle spinning (MAS) on the neat SIC polymer, the LMTO‐1073 sample, and the SIC‐LMTO composite electrolyte, are shown in Figure S8 (Supporting Information). A unique ^6^Li NMR resonance is observed at −1.2 ppm for the neat SIC polymer, and two broad ^6^Li resonances are observed for the LMTO‐1073 nanorods. The spectrum collected on the SIC‐LMTO composite contains multiple resonances that are close to those observed in the ceramic and neat polymer spectra, although an upfield shift of the LLTO resonances is observed, presumably due to changes in the local magnetic field/susceptibility resulting from the presence of a surrounding polymer matrix. Interestingly, the resonance at −0.6 ppm is sharper for the composite electrolyte as compared to pure LMTO nanorods (−0.5 ppm peak maxima) suggesting faster Li‐ion dynamics in the polymer‐ceramic composite. Control samples using P25‐TiO_2_ or commercial LLTO as filler are denoted as SIC‐TiO_2_ or SIC‐LLTO, respectively. The SIC polymer or composite electrolytes were put in the spacer‐free dielectric cells consisting of two symmetric parallel electrode disks and measured by broadband dielectric spectroscopy (BDS) in the frequency range from 1 Hz to 10 MHz and a temperature range from 183 to 353 K. The temperature‐dependent conductivity of all the samples in Figure 5c exhibits Vogel–Fulcher–Tammann (VTF) behavior, indicating the dominant conductivity contribution from the polymer domains.^[^ ^52^ ^]^ As shown in Figure 5c, the conductivity of SIC‐LMTO is the highest among the four electrolytes in the whole temperature range. The filler/polymer interface is controlled by the surface area of the filler. The Brunauer–Emmett–Teller (BET) surface areas of LMTO‐1073 and commercial LLTO were calculated from nitrogen adsorption isotherms. As shown in Figure S9 (Supporting Information), the BET surface area of LMTO‐1073 is 14.3 m^2^ g^−1^, much higher than that of commercial LLTO (0.2 m^2^ g^−1^), while lower than that of P25‐TiO_2_ (24.8 m^2^ g^−1^). At 303 K, the incorporation of LMTO nanorods significantly improves ion transport (SIC‐LMTO), whereas the addition of commercial LLTO microparticles with irregular shapes (Figure S14, Supporting Information) proves ineffective or even detrimental (SIC‐LLTO, Figure 5d). The mechanism underlying the conductivity enhancement in LMTO‐based solid composite electrolytes (SCEs) is further explored in our subsequent work, recently published on Macromolecules.^[^ ^52^ ^]^ The enhanced Li^+^ mobility is attributed to the formation of an interfacial polymer layer around the LMTO nanorods, with a thickness of ≈5 nm. The higher BET surface area of the LMTO nanorods compared to LLTO microparticles (14.3 vs 0.2 m^2^ g^−1^) makes nanorods more efficient at promoting the percolation of the interfacial region. Additionally, while P25‐TiO_2_, used as a control sample, has an even higher surface area (24.8 m^2^ g^−1^), the corresponding SCE (SIC‐TiO_2_) exhibited lower conductivity than SIC‐LMTO, underscoring the advantages of nanorods (Figure 5d). As reported in the literature,^[^ ^22^ ^]^ ceramic nanorods with a high aspect ratio embedded in a polymer matrix can create a 3D ion‐conducting network, enabling long‐range Li^+^ transfer, which is superior to the isolated distribution of nanoparticles within the polymer matrix (Figure S15, Supporting Information). Thus, LMTO NRs with a single‐crystal structure and a high surface area are promising filler materials for advanced SCEs.

The electrochemical performance of SIC‐LMTO was compared to that of the neat SIC polymer through cyclic Li stripping/plating experiments in Li//Li symmetric cells. The Li symmetric cells containing SIC‐LMTO showed much lower overpotential than the SIC cells at current densities from 0.024 to 1 mA cm^−2^ at 343 K (Figure 5e). Figure S10 (Supporting Information) shows the electrochemical profiles of these two cells from 18 to 21 h of cycling. For the SIC cell, the voltage profile is noisy and shows typical soft shorting behavior. In contrast, the cell with SIC‐LMTO electrolyte quickly reached a steady‐state plateau potential of 0.04 V. The plateau is stable and did not show the same soft shorting behavior. The impedance of the Li symmetric cell with SIC and SIC‐LMTO was taken before and after cycling at each current density (Figure S11, Supporting Information). It shows typically depressed semicircles, which correspond to the resistance for ion conduction in the bulk electrolyte and across the Li metal/electrolyte interfaces. Notably, the impedance of the non‐cycled SIC is significantly larger than the non‐cycled SIC‐LMTO (Figure 5f). The resistance of the SIC cell (Figure S11a, Supporting Information), remains relatively unchanged up to cycling at 0.096 mA cm^−2^. The shape of the Nyquist plots changed, and evolved into a single semicircle after cycling at 0.24 mA cm^−2^, accompanied by a sudden drop in impedance to low values, indicating the formation of soft shorts. Whereas the cell made with SIC‐LMTO exhibited no signs of shortage even at a current density of up to 0.48 mA cm^−2^ (Figure S11b, Supporting Information), demonstrating superior plating/stripping performances with reduced overpotential compared to the pure polymer cell. These results show that the addition of LMTO‐1073 NRs to the SIC electrolytes led to lower interfacial impedance with Li metal, increased stability, lower overpotential, and higher rate capability.

We further evaluated the electrochemical window of electrolytes by linear sweep voltammetry (LSV). In Figure S12a (Supporting Information), the oxidative current for pure polymer starts to increase ≈3.6 V versus Li/Li^+^(V), implying the electrolyte starts to decompose. The oxidative current of SIC‐LMTO started to increase at ≈3.9 versus Li/Li^+^(V). SIC‐LMTO showed an improved electrochemical stability window but is relatively limited when considering high‐voltage cathodes such as LiNi_0.6_Mn_0.2_Co_0.2_O_2_ (NMC622). Indeed, when we employed the [Li/NMC622] battery with SIC‐LMTO and conducted a galvanostatic charge/discharge test in the cell voltage range from 2.9 to 4.3 (vs Li/Li^+^(V)) (Figure S12b, Supporting Information), although the cell can be cycled routinely, it exhibits the capacity decay and it still needed further improvements. Due to a power interruption during the first discharge measurement, the cell did not complete the first charge step and a subsequent transition to the second charge step, which subsequently delivered a specific charge capacity of 127 mAh g^−1^. The specific discharge capacity dropped from 170 mAh g^−1^ in the 2nd cycle to 139 mAh g^−1^ by the 5th cycle. This capacity decay can be primarily due to the cut‐off voltage of the cell (2.9–4.3 (vs Li/Li^+^(V)) exceeding the electrochemical stability window of the composite electrolyte. Further investigation into the optimization of electrochemical stability of the electrolyte made with LMTO remains future work, primarily focusing on improving the polymer chemistry.

## 3 Conclusion

In summary, a flux strategy has been developed for the conversion of P25‐TiO_2_ nanoparticles to single‐crystal LMTO nanorods with an entropy stabilized cubic structure. Comprehensive control experiments were performed to clarify the synthetic mechanism and the critical role of flux medium in the growth of single‐crystal LMTO nanorods. The fast Li diffusion in LMTO‐1073 and SIC‐LMTO was proven by solid‐state NMR and BDS tests, respectively. The higher conductivity of SIC‐LMTO than SIC‐LLTO confirms the advantages of the entropy stabilized cubic phase and the nanosized morphology in the conductivity improvement of SCEs. SIC‐LMTO exhibited higher conductivity and lower interface resistance than SIC in Li symmetric cells. This flux synthesis strategy could inspire the synthesis of other single‐crystal nanomaterials such as garnet ceramic electrolytes and perovskite catalysts for improved performance.

## Conflict of Interest

The authors declare no conflict of interest.

## Supporting information



Supporting Information

## Data Availability

The data that support the findings of this study are available from the corresponding author upon reasonable request.
