# Supplementary material for: Flux Synthesis of A‐site Disordered Perovskite La0.5M0.5TiO3 (M═Li, Na, K) Nanorods Tailored for Solid Composite Electrolytes
Source: Adv Sci (Weinh). 2024 Nov 25;12(3):2408805. doi: 10.1002/advs.202408805 (PMC11744714; doi:10.1002/advs.202408805)
Supplement: Supplementary file 1 — Supporting Information [file ADVS-12-2408805-s001.docx]

Supporting Information

Flux Synthesis of A-site Disordered Perovskite La_0.5_M_0.5_TiO_3_ (M = Li, Na, K) Nanorods Tailored for Solid Composite Electrolytes

Tao Wang,* Jiyoung Ock, X. Chelsea Chen, Fan Wang, Meijia Li, Matthew Chambers, Gabriel M. Veith, Lauren B. Shepard, Susan B. Sinnott, Albina Borisevich, Miaofang Chi, Amit Bhattacharya, Raphaële J. Clément, Alexei P Sokolov, and Sheng Dai*

EXPERIMENTAL SECTION

**Materials**

P25-TiO_2_ (30-40 nm) was purchased from Nanostructured & Amorphous Materials Inc. LiNO_3_, La(NO_3_)·6H_2_O were purchased from Sigma-Aldrich. KCl, and NaCl were purchased from Fisher. Commercial LLTO was purchased from Toshima. All chemicals were used as received.

Experimental Section/Methods

*Flux Synthesis of LMTO nanorods*: The reaction mixture was made by mixing P25-TiO_2_: LiNO_3_: La(NO_3_)·6H_2_O: KCl: NaCl at a mass ratio of 1:2:0.3:2.8:2.2 in an agate mortar and ball milled for 5 min in a ZrO_2_ jar. The La:Ti molar ratio difference between target structure and raw material is due to the existence of minor impurities listed in Figure 2d, such as TiO_2_ and La_2_Ti_2_O_7_. The mixture was dried at 473 K for 2 hours in an oven. For the synthesis of LMTO-1073, then the mixture was heated to 1073 K for 5 h with a heating rate of 5 K/min in an alumina crucible under air atmosphere to complete the flux process. After naturally cooling down to room temperature, the salts in the mixture were washed off with water by vacuum filtration. The white cake was dried at 383 K for 2 h in a vacuum oven, hand grinded into powder in an agate mortar, and then heated to 873 K for 2 h with a heating rate of 5 K/min in air to obtain LMTO-1073.

Other LMTO-x products except LMTO-1173 were synthesized under the same condition by changing the flux temperature accordingly. LMTO-1173 and LLTO-1173 were synthesized by increasing the flux temperature to 1173 K for 10 h with a heating rate of 5 K/min.

LLTO-1173 were performed under the same condition of LMTO-1173 by removing NaCl and KCl from the reaction mixture.

*Statistical Analysis*: The SEM and TEM images of LMTO nanorods were open and measured by ImageJ (http://imagej.org) to get the rod width of nanorods. At least 100 nanorods were manually measured for each LMTO sample for size distribution analysis. The rod width data was import in Microsoft Excel spreadsheet to achieve Box and Whisker charts, where the boxes are interquartile ranges and the D50 rod width is marked on each box.

*Synthesis of single-ion-conducting (SIC) polymer and composite electrolytes:*Vinyl ethylene carbonate (VEC, Sigma-Aldrich) was dried over molecular sieves and stored in an Ar glovebox before use. Lithium sulfonyl(trifluoromethane sulfonyl)imide methacrylate (LiMTFSI, SPECIFIC POLYMERS) was stored in an Ar glovebox and used as received. Azobisisobutyronitrile (AIBN, Sigma-Aldrich) was recrystallized in methanol and dried before use. The SIC polymer and composites were prepared via in situ polymerization.^[52]^ First, VEC and LiMTFSI were mixed in a molar ratio of 10:1, followed by adding inorganic fillers (for composites only) and AIBN as the thermal initiator. This was done inside an Ar glovebox. Then the precursor mixture was stirred for 1 hour inside the glovebox (for the SIC polymer) or thoroughly mixed using the Turbula Mixer (GlenMills INC.) for 10 min using yttria-stabilized zirconia balls (for the composite electrolyte). The precursor mixture was then transferred into the measurement cells (dielectric spectroscopy cells, coin cells, or sealed vials) inside the glovebox, which were then sealed in an air-tight mason jar. Polymerization was performed in a temperature-controlled chamber (Heratherm OGH60, Thermo SCIENTIFIC) for overnight at 353 K.

**Characterizations**

TGA/DTA data of the upcycling mixture was collected by STA300, HITACHI, using a heating rate of 5 K/min from room temperature to 1073 K in air. Powder X-ray diffraction (XRD) patterns were collected by a PANalytical Empyrean diffractometer at 45 kV and 40 mA with Cu with a 2:1 mixture of Kα_1_:Kα_2_ (*λ*_1_ = 1.54056 Å, *λ*_2_ = 1.54439 Å) radiation. Inductively coupled plasma-optical emission spectroscopy (ICP) (Thermo Scientific iCAP 7400 ICP-OES Duo) was performed to quantitatively characterize the Li−La−Ti ratio. The procedure to prepare LMTO solutions was performed by following Malkowski et al.^[53]^ and Chambers et al.^[33]^ Approximately 40 mg of material was mixed with a 1:1 volume mixture of HNO_3_:H_2_SO_4_ (10 mL) followed by the slow addition of 2 mL of 30% H_2_O_2_. Reagents were purchased from Fisher Scientific and were Trace Metal-grade or Electronic-grade (H_2_O_2_). Samples were digested in a microwave digestor (CEM-MARS6) at 533 K for 90 min. Care should be taken when conducting this procedure due to the generation of an orange NOx gas. Calibration standards were prepared from 1,000 ppm TraceCERT standards (Aldrich). Scanning electron microscopy (SEM) images were captured on a Zeiss Auriga SEM operated at an accelerating voltage of 5 kV. EDS images were captured on a ZEISS EVO SEM operated at an accelerating voltage of 15 kV. Transmission electron microscopy (TEM) images were captured on a Fisher Scientific Spectra 300 operated at an accelerating voltage of 200 kV. ^7^Li NMR experiments were performed on a Solid-State NMR Varian INOVA 400 MHz, equipped with a 5 mm Chemagentic CPMAS probe. ^7^Li static spectra were acquired with a single pulse experiment at a Larmor frequency of 155.38 MHz, with a 90° pulse of 5 μs and a recycle delay of 3.0 s. Both ^6^Li and ^7^Li chemical shifts were referenced to the Li signal of a LiCl solution. All spectra were simulated with DMFIT software utilizing the G/L mode. All solid-state one-dimensional ^6^Li MAS-NMR spectra were collected at 18.8 T on a Bruker AVANCE standard bore III Ultrashield Plus spectrometer using a 2.5 mm H-X magic angle spinning (MAS) probe with 2.5 mm (O.D.) zirconia rotors which were spun at 30 kHz. All the samples were packed into the rotors inside the Ar-filled glove box. ^6^Li data were acquired with a π/2 pulse of 4 µs, recycle delay of 40 s, and referenced to a 1 M LiCl aqueous solution at 0 ppm. NMR spectra were processed using the Bruker TOPSPIN 4.3.0 software and 1-10 Hz line broadening. ABF STEM images were recorded using Nion® UltraSTEM 200 aberration-corrected scanning transmission electron microscope operated at 200 kV. LMTO-800 samples were prepared from powders by embedding in Araldite epoxy resin (to access orientations other than normal to long nanorod axis) and sectioning with Leica EM UC7 Ultramicrotome at room temperature.

**Broadband dielectric spectroscopy measurement**

The conductivity of SIC polymer and composite electrolytes was measured with a broadband dielectric spectroscopy (BDS) using an Alpha–A spectrometer (Novocontrol) in the frequency range from 10 MHz to 1 Hz. The samples were prepared between two parallel electrode disks of 10.2 mm in diameter and separated by 0.3 mm in a spacer-free dielectric cell. The samples were placed inside the cryostat in a nitrogen atmosphere. Before measurement at each temperature, the sample was equilibrated to reach thermal stabilization within 0.2 K after each temperature step. Measurements were conducted from low to high temperatures and recorded upon heating.

**Electrochemical measurements**

**Linear sweep voltammetry test.** All electrochemical measurements were performed at 343 K. The electrochemical stability of SIC and SIC-LMTO were evaluated using linear sweep voltammetry (LSV) with two electrode cells. LSV was performed on Mo foil as working electrode, Li metal chip (MTI Corporation) as both counter and reference electrode, at a scan rate of 0.05 mV/s potential range from open circuit voltage (OCV) to 6 V (vs Li/Li^+^(V)).

**Battery test.** Li stripping/plating tests were conducted on a Li symmetric cell. Li metal chips were used as received. 100 µL of the polymer or composite precursor mixture was spread onto a glass fiber separator placed between two Li metal chips and assembled in 2032-type coin cell in an argon-filled glove box and subsequently in situ polymerized in a temperature chamber. After polymerization, the Li symmetric cells were further conditioned at 343 K for a day and cycling measurements were carried out using a potentiostat/galvanostat (Biologic, VMP3) at varying current densities of 0.024, 0.048, 0.096, 0.24, 0.48 and 1 mA cm^-2^ for five cycles at each current density. After each cycling test, electrochemical impedance spectroscopy (EIS) was conducted in the frequency range of 1 MHz to 100 mHz with a voltage amplitude of 6 mV.

Galvanostatic charge/discharge measurements were conducted on [Li metal chip/SIC-LMTO/NMC622 cathode] cell. The cathode was composed of NMC 622 (90 wt %, BASF), carbon black (5 wt %, Denka), and poly(vinylidene fluoride) (5 wt %, Solvay). The dried cathode sheet was punched into a circular shape with a diameter of 7/16 discs and the typical mass loading of the active material in the cathode was around 12.5 mg/cm^2^. The cells were aged at 343K for a day before testing. Galvanostatic charge-discharge tests were performed using an electrochemical analyzer in the potential range of 2.9–4.3 V. The charge and discharge current density was fixed at 0.049 mA/cm^2^ for 5 cycles.

**Rietveld Refinement**

Rietveld refinement^[54]^ was performed using TOPAS v7^[55]^ in order to determine phase composition. Backgrounds were modelled using a 12-fold Chebyshev polynomial. A single Thompsons-Cox-Hastings pseudo-Voigt function was applied to all included phases to model peak shapes. Cell parameters of each phase included in the refinement were allowed to refine following symmetry constraints. In preliminary refinements, a single isotropic atomic displacement parameter (ADP) was used for each phase. The atomic site occupancies were not refined. For pseudo-cubic LLTO (space group: *P*4/*mmm*), the starting model determined by Catti *et al*.^[56]^ was used. For cubic LLTO (space group: *Pm*-3*m*), initially the structure determined by Sotomayor *et al*.^[57]^ was modified so that the stoichiometry was fixed to Li_0.3_La_0.57_TiO_3_. In later refinements, Na and K were placed on the 1*b* site (½, ½, ½) along with Li and La to match the stoichiometries determined by ICP. In the final refinements, the occupancies of Li, Na, K, Ti and O were fixed, but the occupancy of the La site was refined. During this refinement, for the LMTO phase, a single ADP was used for the A-site (*i.e*. Li, Na, K and La), while separate ADPs were used for Ti and O. Refinements were also performed using the *R*-3*c* Li_0.2_Na_0.27_La_0.5_TiO_3_ structure determined by Varez *et al*.^[58]^ Additionally, the models for TiO_2_ determined by Baur *et al*.^[59]^ and La_2_Ti_2_O_7_ determined by Schmalle *et al*.^[60]^ were used as secondary phases.

As the pseudocubic polymorph and cubic polymorphs have very similar structures, there can be difficulty in resolving which polymorph is present, especially when there are broad peaks, and a non-monochromatic emission profile is used, as the broadness of the peaks means that the peak splitting characteristic of a tetragonal unit cell results in apparently a single peak and the peaks resulting from Cu Kα_1_ and Kα_2_ radiation can overlap. In order to determine which phases were present, Rietveld refinement was performed using the pseudo-cubic LLTO polymorph^[56]^ and a cubic polymorph.^[57]^ These refinements were based on a formula of La_0.57_ Li_0.3_TiO_3_. In the LMTO-1073 nanorods, both phases could be fit well against the data. When using the pseudo-cubic *P*4/*mmm* polymorph, a fit was obtained with R_wp_ = 4.880% and χ^2^ = 1.484. On the other hand, refining with the cubic *Pm*-3*m* phase resulted in a fit of R_wp_ = 5.084% and χ^2^ = 1.545. However, ICP results show that the atomic ratio of Li:Na:K:La:Ti is approximately 0.11(1):0.243(5):0.02(1):0.431(7):1 in LMTO-1073, indicating Na and K doping in the A sites of LLTO structure. Therefore, a refinement was performed by adding Na and K to the (½, ½, ½) position with a formula of Li_0.11(1)_Na_0.243(5)_K_0.02(1)_La_0.431(7)_TiO_2.82(2)_. The O content was calculated through charge balancing of the cations. This resulted in a fit of R_wp_ = 4.994%, χ^2^ = 1.518. The increase in R_wp_ is due to the lower La content, as the ICP ratio includes the impurity phases present. La is the strongest X-ray scatterer in LMTO and will therefore dominate the X-ray diffraction pattern. Therefore, the La occupancy was allowed to refine while keeping the other A site occupancies fixed due to lack of sensitivity. As ADPs are highly correlated to site occupancies, each atomic site was given its own ADP, with the A-site atoms sharing an ADP. The resulting fit had values of R_wp_ = 4.841% and χ^2^ = 1.473 and an overall composition of Li_0.11_Na_0.24_K_0.02_La_0.49 (1)_TiO_2.831_. On the other hand, Li_0.2_Na_0.27_La_0.5_TiO_3_ has been reported^[58, 61]^ to adopt *R*-3*c* symmetry at room temperature, so a refinement was performed using this structure. A fit was obtained with R_wp_ = 4.961% and χ^2^ = 1.508. Despite having a lower R_wp_, the additional supercell reflections from the rhombohedral cell overlap with each other due to broadness of the peaks. Additionally, Varez *et al*.^[58]^ reported that a cubic phase could be fit against X-ray data, where the rhombohedral supercell reflections were only visible using neutron data. Furthermore, this sample also has a small amount of K present. It has been shown that increasing the entropy of a system by increasing the number of elements on the same site can stabilize high-symmetry phases,^[43-44]^ such as with disordered rock salts^[45]^ and with other perovskites.^[46]^ Therefore, although we cannot exclude the possibility that the LMTO-1073 is present as the pseudo-cubic or rhombohedral polymorphs, it is likely that the LMTO-1073 is present as the cubic *Pm*-3*m* perovskite phase. The with the cubic polymorph with Li, Na, K, and La at A sites described above, also modelled the size broadening of the LMTO crystallites was using a Voigt function.^[47]^ To decrease the La_2_Ti_2_O_7_ impurity in LMTO, the flux temperature was increased from 1073 to 1173 K and the product was named as LMTO-1173. The same Rietveld refinement procedure was performed for LMTO-1173. In this case, it was determined that there is no La_2_Ti_2_O_7_, so only LLTO and TiO_2_ were used in these refinements. ICP analysis indicated that the atomic ratio of Li:Na:K:La:Ti is approximately 0.01:0.27:0.01:0.38:1 in LMTO-1173. Initially refining using the stoichiometry obtained from ICP, resulted in a fit of R_wp_ = 5.481%, χ^2^ = 1.675, where the increase in R_wp_ once again is caused by the difference in La^3+^ content within the LMTO sample and the overall composition. The La^3+^ site occupancy was allowed to refine in the same manner as with LMTO-1073, resulting in a stoichiometry of Li_0.01_Na_0.27_La_0.51 (1)_TiO_2.71_, a crystallite size of 56(5) nm, R_wp_ = 4.607% and χ^2^ = 1.425. The Rietveld plot is shown in Figure S2b. For the same reasons given above, it is most likely that LMTO-1073 and LMTO-1173 are present as the cubic phase.

ICP-derived composition of LMTO-1073 is Li_0.11(1)_Na_0.243(5_)La_0.431(7)_ K_0.02(1)_TiO_2.823(1)_. From the Rietveld refinement, the phase composition of LMTO-1073 is 8.1(6)% TiO_2_, 9.0(5)% La_2_Ti_2_O_7_ and 82.8(8)% LMTO. ICP determines the whole composition, so using these approximate percentages from Rietveld refinement, there is 8×1 + 9×2 = 26% Ti and 9×2 = 18% La outside of LMTO phase. This means that the amount of Ti in LMTO phase is (1–0.26)×1 and the La is (1–0.18)×0.431, giving a new formula of Li_0.109_Na_0.243_La_0.353_K_0.02_Ti_0.74_O_3–x_. Renormalizing this against Ti (i.e. dividing whole composition by 0.74) gives a composition of Li_0.15(1)_Na_0.328(7)_La_0.477(9)_K_0.03(1)_TiO_2.97(2)_ for LMTO phase in LMTO-1073. This formula agrees very well with the Rietveld refinements, where the La content of 0.491(13) is within estimated standard deviation (σ).

Following the control synthesis, additional Rietveld refinements were performed on LLTO-1173 (Figure S6). As the (0 0 1) reflection that arises from the primitive tetragonal unit cells is apparent, refinements were performed with both a *P*4/*mmm* Li_0.5­_La_0.5_TiO_3_ phase based on the model obtained by Ibarra *et al*.^[62]^ and a *P*4/*nbm* Li_0.3_La_0.567_TiO_3_ phase based on the model determined by Catti *et al*.^[63]^ along with the following secondary phases: Li_4_Ti_5_O_12_ (Li *et al*.^[64]^), La_2_Ti_2_O_7_, TiO_2_ (rutile) and LaOCl (Hölsä *et al*.).^[65]^ Separate refinements using the two different tetragonal supercell LLTO phases were performed as it has been shown that in the synthesis of LLTO, different precursors can result in the formation of different polymorphs.^[33]^ Furthermore, several of the secondary phases reported here, such as Li_4_Ti_5_O_12_ and La_2_Ti_2_O_7_, are commonly reported and are likely to form due their high thermal dynamic stability. Refinements with the double-perovskite Li_0.5_La_0.5_TiO_3_ resulted in a fit of R_wp_ = 5.910%, χ^2^ = 1.784. On the other hand, refinements with the *P*4/*nbm* phase resulted in a fit of R_wp_ = 5.644%, χ^2^ = 1.644. When both phases were included, the refinements gave a weight percentage of 9.0(5)% *P*4/*mmm* and 48.1(13)% *P*4/*nbm* and a fit of R_wp_ = 5.339%, χ^2^ = 1.613. However, all the peaks that the *P*4/*mmm* was fitting against were also fit by *P*4/*nbm*, indicating that the LLTO is most likely present as the *P*4/*nbm* polymorph. The final resulting composition is 56.0(6)% *P*4/*nbm* LLTO, 19.1(6)% Li_4_Ti_5_O_12_, 13.7(4)% La_2_Ti_2_O_7_, 6.2(5)% TiO_2_ (rutile) and 4.9(3)% LaOCl. To our knowledge, there have been no reports of an LLTO composition of Li_0.5_La_0.5_TiO_3_ that adopts the *P*4/*nbm* structure; however, due to various secondary phases present, the LLTO composition seen here likely does not have this stoichiometry. We can conclusively demonstrate that neither the cubic nor pseudocubic LLTO phases have formed, revealing that doping of Na^+^ and K^+^ improved the stability of the cubic LLTO phase and prevented additional impurities from forming. The occupancy of La was not refined against this data, as the large number of secondary phases will make it difficult to obtain reasonably accurate numbers. Furthermore, this refinement was performed to demonstrate that no cubic-LLTO was obtained.

**Density Functional Theory Calculations**

The density functional theory (DFT) calculations were done with the plane-wave basis and projector augmented wave (PAW) potentials as implemented in the Vienna Ab initio Simulation Package (VASP).^[66-67]^ A plane wave energy cutoff of 680 eV and Gaussian smearing of 0.05 eV was used for all calculations. The r^2^SCAN meta-generalized gradient approximation was selected for the exchange functional.^[68-69]^ Full geometry optimizations were done for all LMTO structures and compositions, with energy convergence between self-consistent cycles less than 10^-5^ eV and a force convergence criterion of 0.02 eV/Å was used. A $\Gamma$-centered Monkhorst Pack k-point mesh of 8 × 8 × 8, 6 × 6 × 4, 8 × 8 × 4, and 8 × 8 ×8 was used for the cubic, orthorhombic, and pseudocubic perovskite unit cells respectively and scaled accordingly depending on supercell size needed to achieve wanted Na and/or K compositions. Special quasirandom structures (SQS) of La_0.5_Li_0.5–_*_x_*M*_x_*TiO_3_ (M = Na and/or K) were generated for composition, *x*, values ranging from 0 to 0.375 with the integrated cluster expansion toolkit (ICET).^[10, 70]^ To generate the LMTO SQS, structure files for perovskite LaTiO_3_ in the cubic (*Pm*-3*m*), and orthorhombic (*Pnma*) phases were obtained from the Materials Project database and the structure file for La_0.567_Li_0.3_TiO_3_ was obtained from Inorganic Crystal Structure Database (ICSD).^[71-72]^

$$t=\frac{r_{A}+r_{O}}{\sqrt{2}\left( r_{Ti}+r_{O} \right)}$$

Tolerance factors were calculated using the above equation for various Na and/or K doping compositions in LMTO.

**Figure:**

**Figure S1.** TG and DTA curves of the reaction mixture for the synthesis of LMTO-1073.


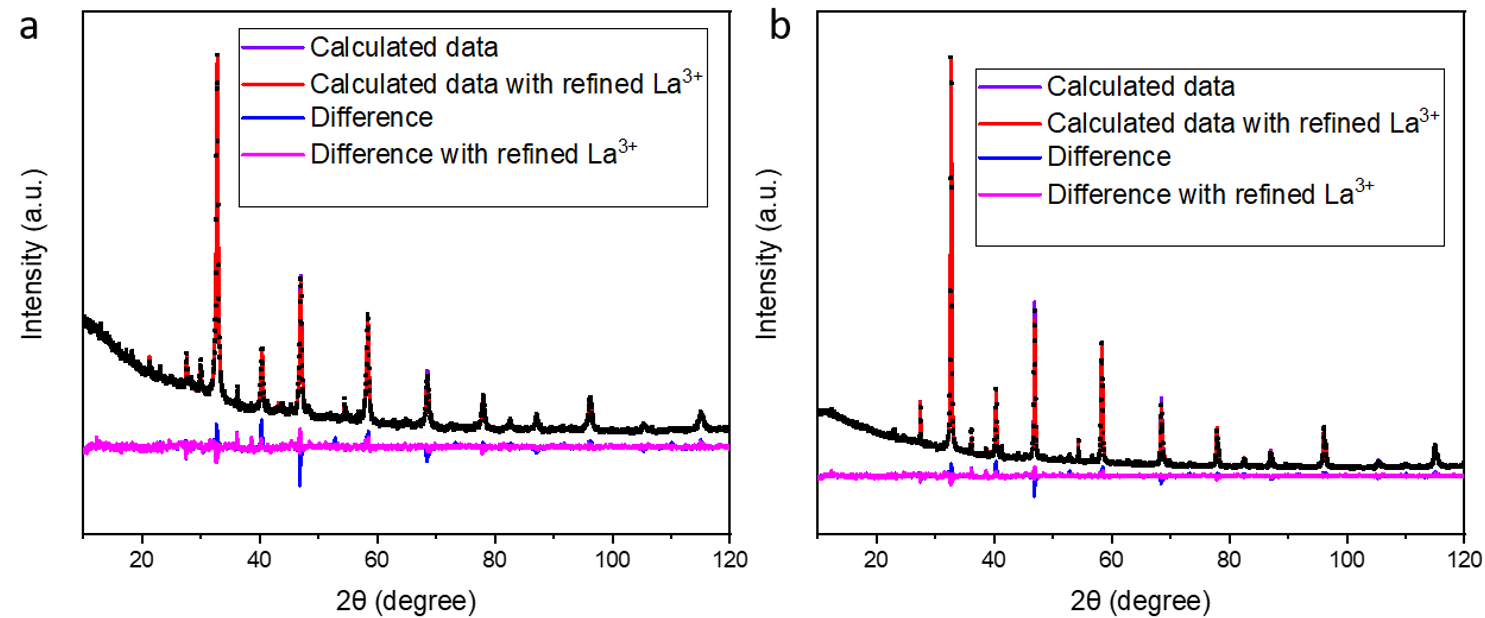


**Figure S2.** Rietveld plots obtained from (a) LMTO-1073 and (b) LMTO-1173.

**
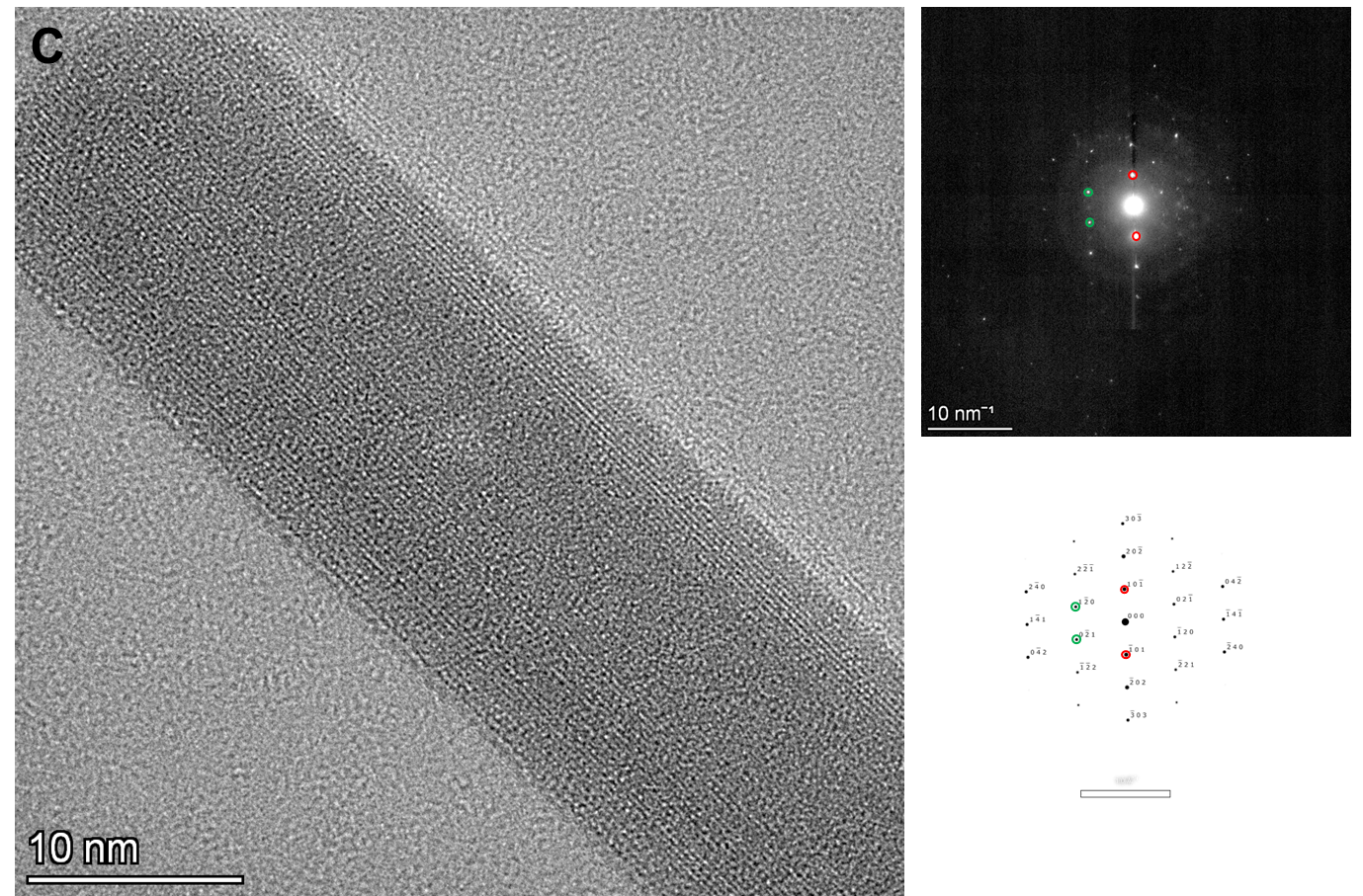
**

(221),

Pm3m

**Figure S3.** (a-b) ABF STEM images of LMTO-1073 nanowires and diffractograms of image areas highlighted in red alongside the best match for closest crystal orientation. (c)TEM image and SAED pattern of an LMTO-1073 nanowire alongside the best match for closest crystal orientation. Matching reflections in simulated patterns and experimental diffractograms are highlighted by circles of the same color. Diffraction patterns were simulated using CrystalMaker Software Ltd, Oxford, England, SingleCrystal for cubic La_0.5_Li_0.5_TiO_3_^[73]^. Scale bars on experimental and simulated patterns are the same length, 5 nm^-1^ for (a,b) and 10 nm ^-1^ for (c).


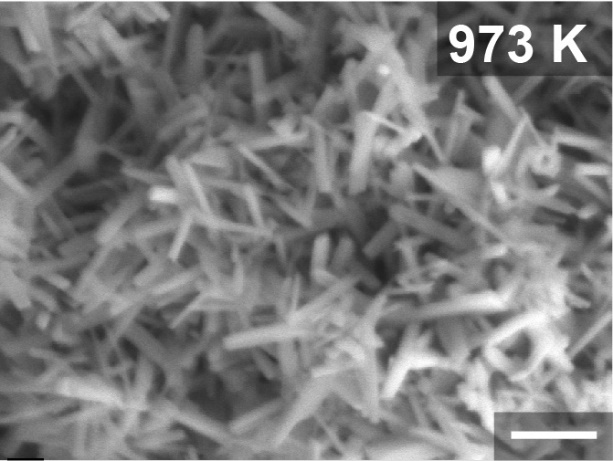


**Figure S4.** SEM image of LMTO-973.


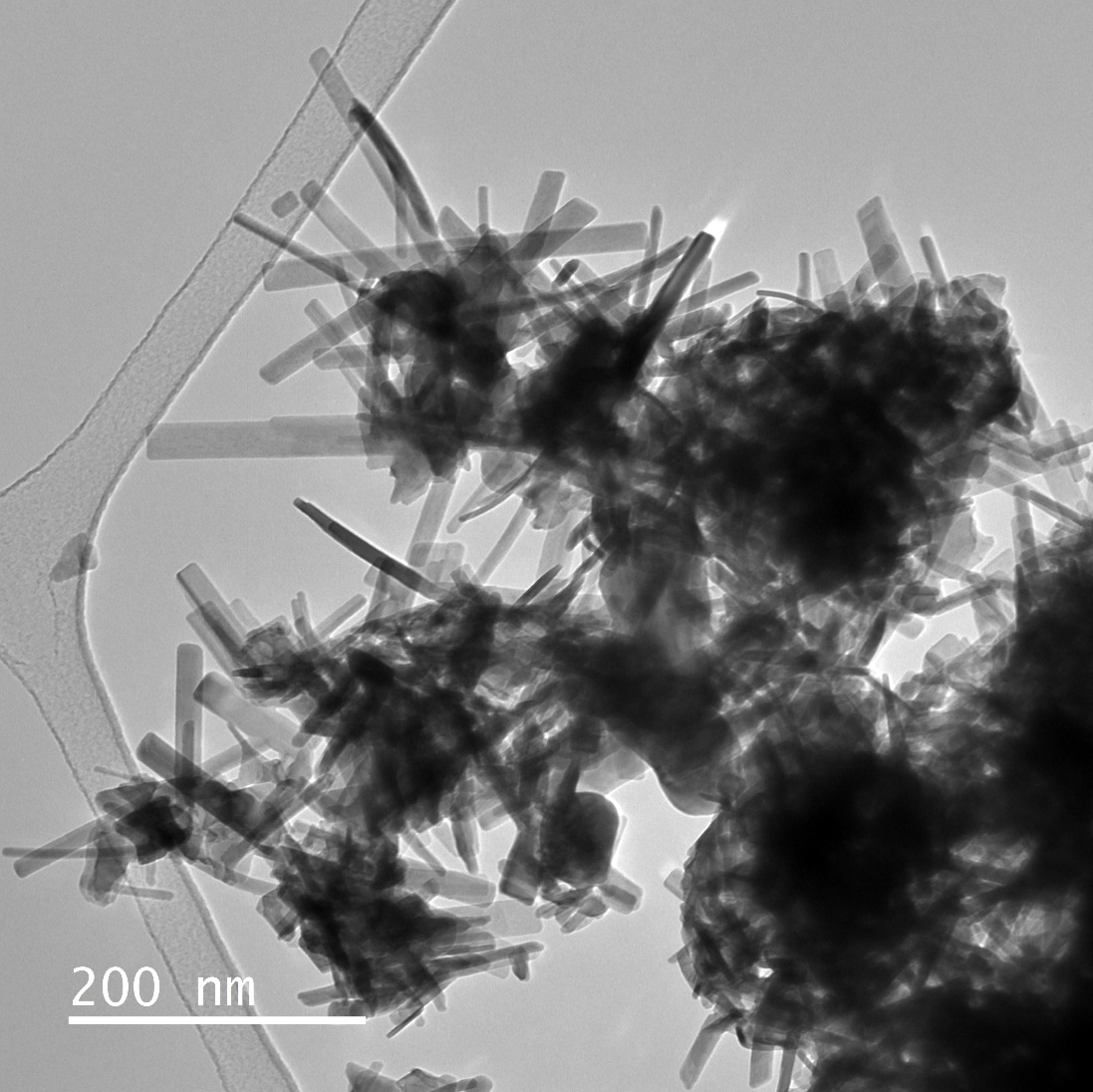


**Figure S5.** TEM image of LMTO-773.


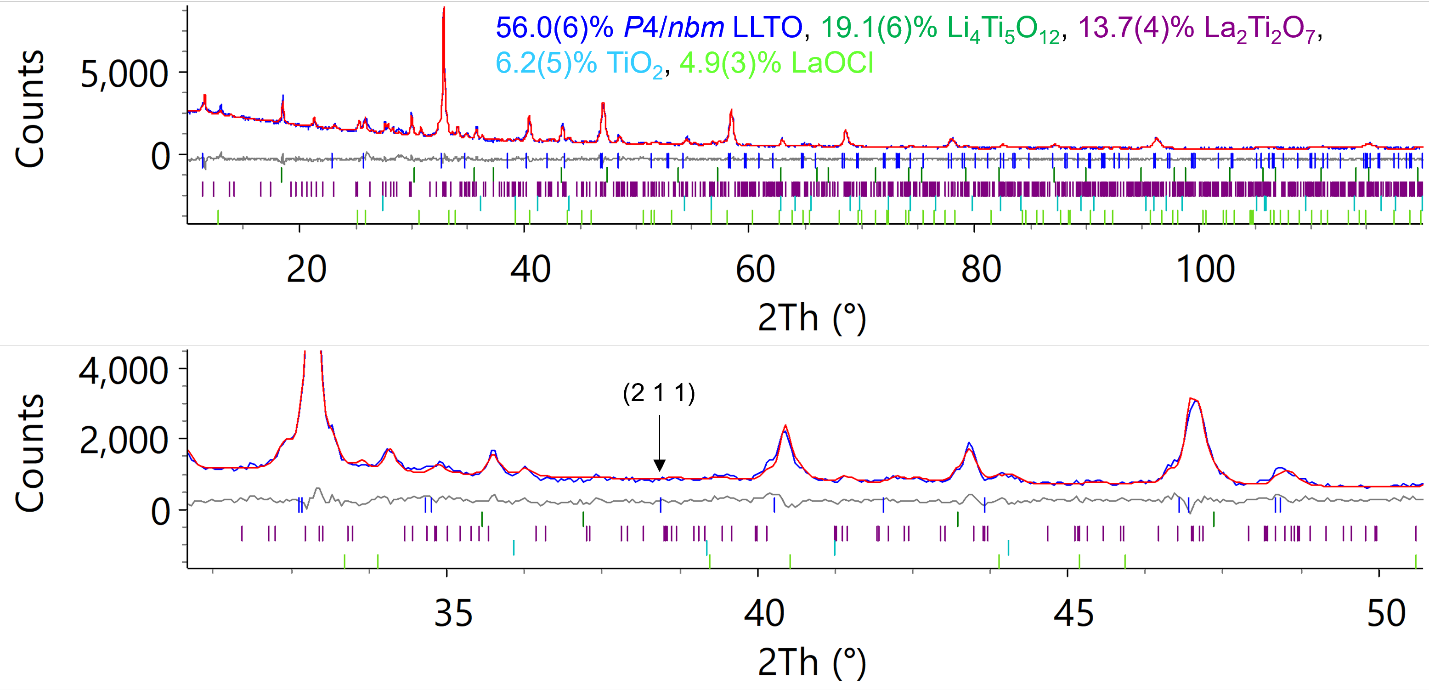


**Figure S6.** Rietveld plots obtained from LLTO-1173.

**Figure S7.** Average Ti-O-Ti bond angle with varying M compositions obtained from DFT geometry relaxations of orthorhombic (Pnma) phase of La_0.5_Li_0.5-x_M_x_TiO_3_ where M is potassium/K (black), sodium/Na (red), and both Na and K in equal compositions (blue).


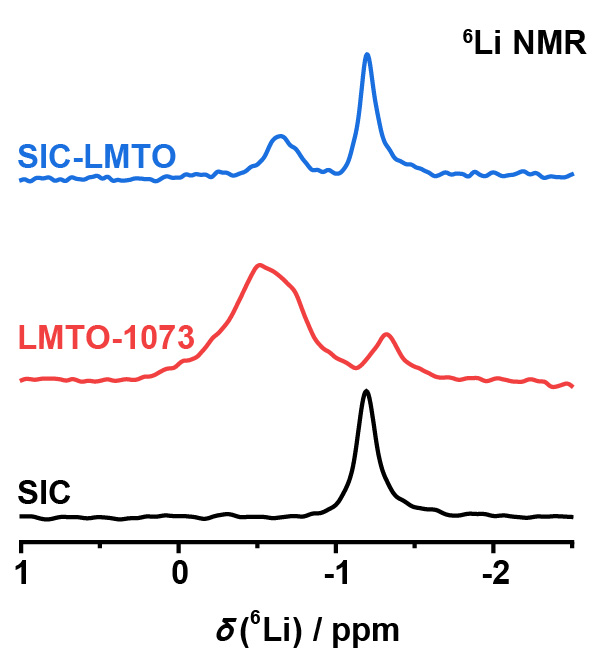


**Figure S8.** ^6^Li Hahn echo NMR spectra collected on SIC, LMTO-1073, and the SIC-LMTO composite at 18.8 T, 30 kHz MAS and 303 K.

**Figure S9.** N_2_ adsorption isotherms of LMTO-1073, P25-TiO_2_, and commercial LLTO.


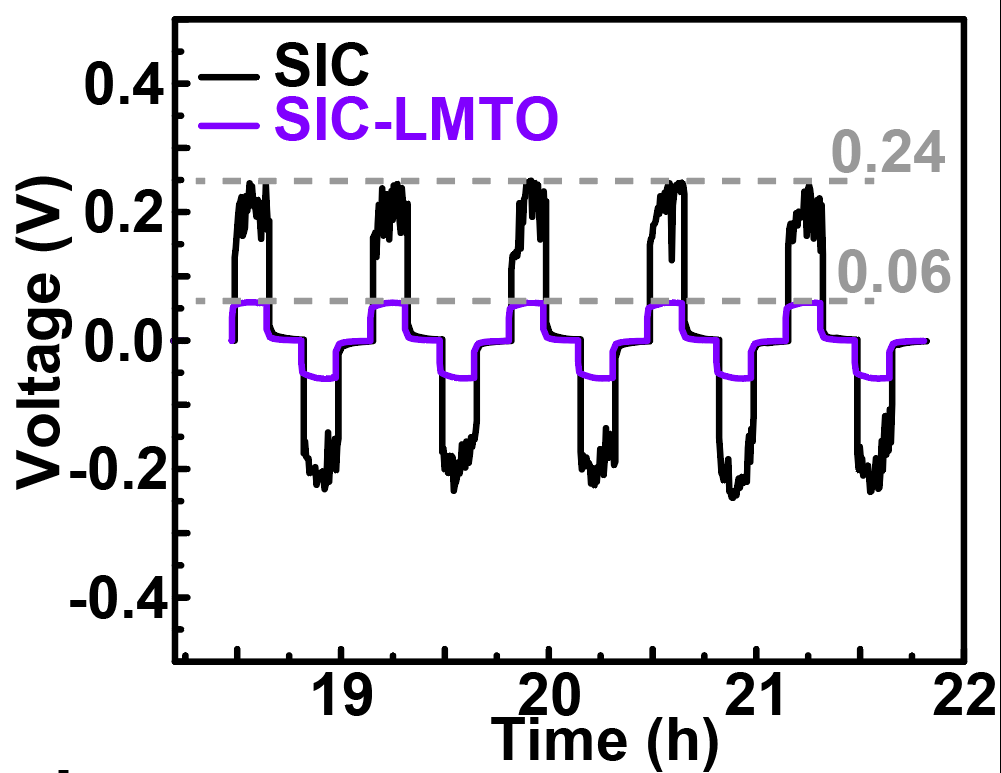


**Figure S10.** Li symmetrical cell cycling profiles at 0.48 mA/cm^2^.

**Figure S11.** (a) The Nyquist plots of the Li symmetric cell made with SIC and (b) SIC-LMTO at open circuit voltage (OCV, before cycling) and after stripping/plating at current densities of 0.024, 0.048, 0.096, 0.24, 0.48 and 1 mA/cm^2^ at 70 °C.

**Figure S12.** (a) Linear sweep voltammograms of SIC and SIC-LMTO at a scan rate of 0.05 mV/s at 70 °C. (b) Charge/discharge curves of Li/SIC-LMTO/NMC622 measured at charge/discharge current density of 0.049 mA/cm^2^.


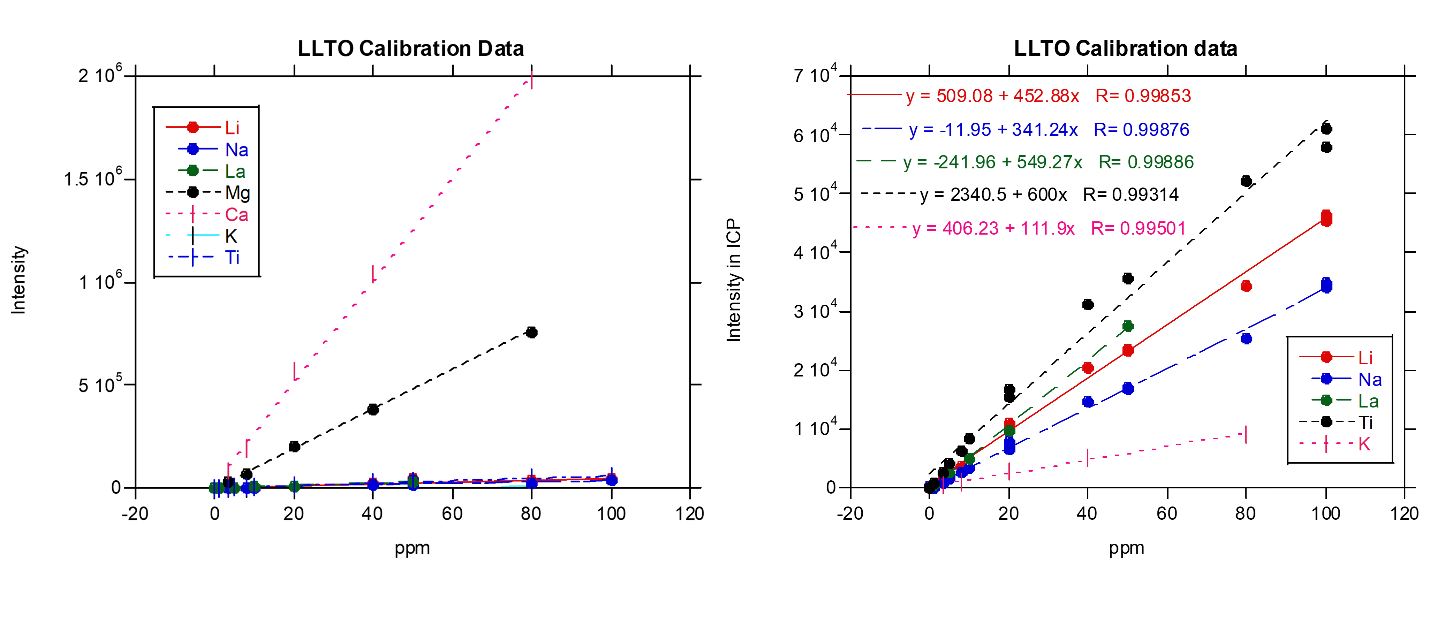


**Figure S13.** Calibration data of ICP-OES tests.


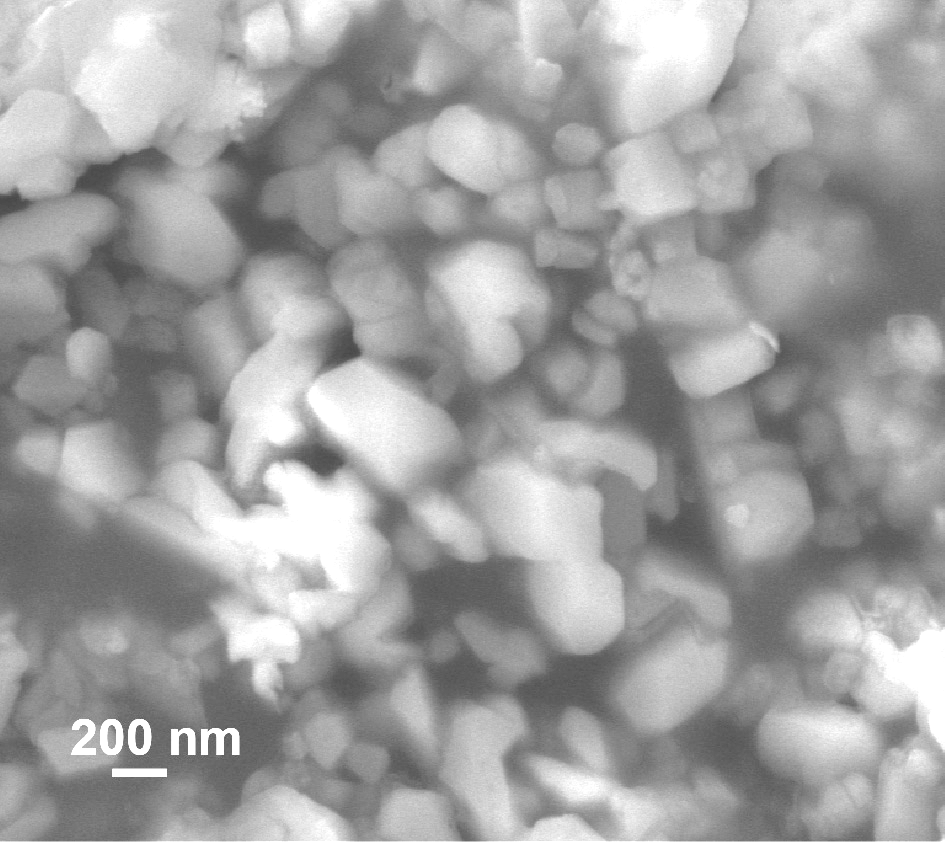


**Figure S14.** SEM image of commercial LLTO microparticles.


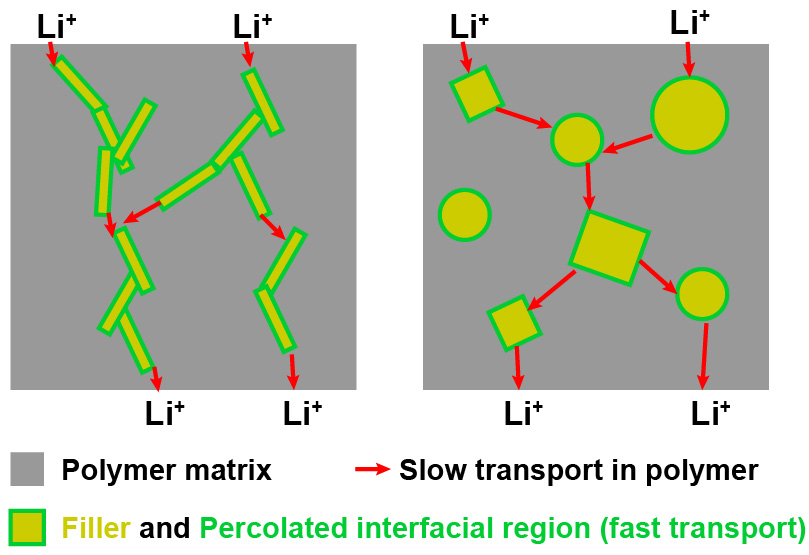


**Figure S15.** Illustration of the effect of filler morphology on Li^+^ ion transport in SCEs.

**Table S1.** Mole ratios of Li, Na, K, and La to Ti according to ICP results.

| **Samples** | **Moles Li** | **Moles Na** | **Moles K** | **Moles La** | **Moles Ti** |
| --- | --- | --- | --- | --- | --- |
| LMTO-1073 | 0.11 (1) | 0.243(5) | 0.02(1) | 0.431(7) | 1 |
| LMTO-1173 | 0.01 (1) | 0.267(5) | 0.01(1) | 0.381(7) | 1 |
| LLTO-1173 | 0.32 (1) | 0.013(5) | 0 | 0.379(7) | 1 |

**REFERECE**

[53] T. F. Malkowski, E. D. Boeding, D. Fattakhova-Rohlfing, N. Wettengl, M. Finsterbusch, G. M. Veith, *Ionics* **2022**, *28*, 3223-3231.

[54] H. Rietveld, *Journal of Applied Crystallography* **1969**, *2*, 65-71.

[55] A. A. Coelho, J. Evans, I. Evans, A. Kern, S. Parsons, *Powder Diffraction* **2011**, *26*, S22-S25.

[56] M. Sommariva, M. Catti, *Chemistry of Materials* **2006**, *18*, 2411-2417.

[57] M. E. Sotomayor, B. Levenfeld, A. Varez, J. Sanz, *Journal of Alloys and Compounds* **2017**, *720*, 460-465.

[58] A. Varez, M. T. Fernandez-Díaz, J. Sanz, *Journal of Solid State Chemistry* **2004**, *177*, 4665-4671.

[59] W. H. Baur, A. A. Khan, *Acta Crystallographica Section B* **1971**, *27*, 2133-2139.

[60] H. W. Schmalle, T. Williams, A. Reller, A. Linden, J. G. Bednorz, *Acta Crystallographica Section B* **1993**, *49*, 235-244.

[61] R. Jimenez, A. Rivera, A. Varez, J. Sanz, *Solid State Ionics* **2009**, *180*, 1362-1371.

[63] J. Ibarra, A. Várez, C. León, J. Santamarıa, L. M. Torres-Martınez, J. Sanz, *Solid State Ion.* **2000**, *134*, 219-228.

[63] M. Catti, M. Sommariva, R. M. Ibberson, *Journal of Materials Chemistry* **2007**, *17*, 1300-1307.

[64] C. Li, J. Mao, *Journal of Solid State Chemistry* **2021**, *296*, 121974.

[65] J. Hölsä, M. Lastusaari, J. Valkonen, *Journal of Alloys and Compounds* **1997**, *262-263*, 299-304.

[66] G. Kresse, J. Furthmüller, *Phys. Rev. B* **1996**, *54*, 11169.

[67] G. Kresse, D. Joubert, *Phys. Rev. B* **1999**, *59*, 1758.

[68] S. P. Ong, W. D. Richards, A. Jain, G. Hautier, M. Kocher, S. Cholia, D. Gunter, V. L. Chevrier, K. A. Persson, G. Ceder, *Comput. Mater. Sci.* **2013**, *68*, 314-319.

[69] J. W. Furness, A. D. Kaplan, J. Ning, J. P. Perdew, J. Sun, *J. Phys. Chem. Lett.* **2020**, *11*, 8208-8215.

[70] A. Zunger, S. H. Wei, L. G. Ferreira, J. E. Bernard, *Phys. Rev. Lett.* **1990**, *65*, 353.

[71] A. Jain, S. P. Ong, G. Hautier, W. Chen, W. D. Richards, S. Dacek, S. Cholia, D. Gunter, D. Skinner, G. Ceder, *APL Mater.* **2013**, *1*.

[72] M. Sommariva, M. Catti, *Chem. Mater.* **2006**, *18*, 2411-2417.

[73] J. Ibarra, A. Várez, C. León, J. Santamarıa, L. M. Torres-Martınez, J. Sanz, *Solid State Ion.* **2000**, *134*, 219-228.
